# If These Walls Could Talk: In-home Activity and Relationship Ratings among Older Adult Couples at Risk for Dementia

**DOI:** 10.1093/geroni/igaf122.2934

**Published:** 2025-12-31

**Authors:** Lyndsey Anderson, Joel Steele, Wan-Tai Au-Yeung, Thomas Riley, Victoria Sanchez, Zachary Beattie, Jeffrey Kaye

**Affiliations:** Oregon Health & Science University, Portland, Oregon, United States; University of North Dakota, Grand Forks, North Dakota, United States; Oregon Health & Science University, Portland, Oregon, United States; Oregon Health & Science University, Portland, Oregon, United States; Oregon Health & Science University, Portland, Oregon, United States; Oregon Health & Science University, Portland, Oregon, United States; Oregon Health & Science University, Portland, Oregon, United States

## Abstract

Functional decline related to Alzheimer’s disease is an interdependent process among older adult couples due to mutual health behaviors, as well as shared stressors and daily activities. The objective of this study was to examine variability in activity in the home among older adult couples that include a partner who is at high-risk of developing AD, and relate levels of variability in activity to perceived spousal support. Data were derived from the study: Remote Sensing of (older adult partners’) Engagement in Life and Variability in Everyday Support (RSELVES), which is prospective longitudinal and uses home-based assessment to continuously generate digital evaluations of engagement in life and weekly dyadic support appraisals among couples . Multiple in-home sensors were used to create a whole house activity state array (WHASA), encoding minute level activity over the day, that was subsequently modeled along with weekly self-reported measures of perceived spousal support (Secure Base Characteristics Scale). Activity entropy was aggregated hourly to capture trends in daily activity variability. Results showed that polynomial coefficients of variability were significantly associated with differences between partners’ support ratings (encouragement, availability, and intrusiveness) and average support levels. Higher in-home activity variability correlated with greater incongruence in couples’ weekly reports of encouragement and availability. Additionally, higher average encouragement levels related to later-day peaks in activity variability. The WHASA approach effectively captured patterns of dyadic home activity and demonstrated relationships with older couples’ perceptions of partner support, indicating interdependence in both functional and relational aspects of life engagement.

